# Reproductive-Age Women's Knowledge and Care Seeking for Malaria Prevention and Control in Ghana: Analysis of the 2016 Malaria Indicator Survey

**DOI:** 10.1155/2019/2316375

**Published:** 2019-02-12

**Authors:** Martin Amogre Ayanore, John Tetteh, Asiwome Ameko, Wisdom Kudzo Axame, Robert Kaba Alhassan, Augustine Adoliba Ayanore, Victor Mogre, Seth Owusu-Agyei

**Affiliations:** ^1^Department of Family and Community Health, School of Public Health, University of Health and Allied Sciences, Hohoe campus, Ghana; ^2^Centre for Health Policy Advocacy, Innovation & Research in Africa (CHPAIR-Africa), Ghana; ^3^Department of Epidemiology and Biostatistics, School of Public Health, University of Health and Allied Sciences, Hohoe campus, Ghana; ^4^School of Nursing and Midwifery, University of Health and Allied Sciences, Ho, Ghana; ^5^Department of Epidemiology and Biostatistics, School of Public Health, University of Ghana, Accra, Ghana; ^6^Department of Health Professions Education and Innovative Learning, School of Medicine and Health Sciences, University for Development Studies, Tamale, Ghana; ^7^Institute of Health Research, University of Health and Allied Sciences, Ho, Ghana

## Abstract

**Introduction:**

Malaria is a major cause of morbidity and mortality worldwide, requiring individual and environmental level controls to prevent its adverse morbidity effects. This study examined reproductive-aged women's knowledge and care-seeking practices for malaria prevention and control in Ghana.

**Methods:**

The 2016 Ghana Malaria Indicator Survey data for reproductive-age women was analysed (n=5,150). Multilevel mixed-effects logistic regression model was used to determine factors associated with reproductive-aged women's knowledge and care-seeking practices for malaria.

**Results:**

62.3%, 81.3%, and 64.6% knowledge levels on causes, signs/symptoms, and prevention of malaria were found, respectively, among respondents. Age, wealth and educational status, religion, region, and place of residence (rural) were found to significantly influence respondents' knowledge of causes, signs/symptoms, and care-seeking practices for malaria. A 15% differential among Insecticide Treated Nets (ITNs) awareness and use was found. Increasing age (≥35 years) was associated with increasing knowledge of malaria. Regional variations were observed to significantly influence knowledge of malaria treatment.

**Conclusion:**

Though ownership of ITNs and knowledge of malaria prevention were high, it did not necessarily translate into use of ITNs. Thus, there is a need to intensify education on the importance and the role of ITNs use in the prevention of malaria.

## 1. Introduction

In Ghana, malaria is a major cause of death and other socioeconomic losses due to morbidity and social, economic, and health implications [1–5]. Aggregate estimates from the district health information data show that about 2000 deaths in Ghana are attributable to malaria, with approximately 48% of these case fatalities affecting children under 5 years [6].* Plasmodium falciparum* accounts for 80-90% of malaria reported morbidity issues in Ghana, particularly among pregnant women and children under 5 years [7]. The National Malaria Control Programme (NMCP) which became operational in 1999 sets up the modalities to reduce the burden of malaria nationally [8].

Malaria control interventions such as the introduction of Insecticide Treated Nets (ITNs), Indoor Residual Spraying (IRS), and Rapid Diagnostic Tests (RDTs) which form part of key strategies in Ghana have contributed significantly to malaria control in Ghana [9]. World Health Organization (WHO) advocates a mix of strategies to combat malaria, from an increased awareness of malaria causes and prevention, improvements in surveillance, and advances in new vaccine control mechanisms [10]. A new strategic plan (2015-2020) against malaria in Ghana provides a renewed framework to reduce malaria-related mortality rates by 75% in 2020 [2, 9, 11].

Pregnant women and children under 5 years remain vulnerable in the epidemiology of the malaria disease worldwide. According to national estimates from the 2016 Ghana Malaria Indicator Survey (GMIS) report, about 54.3% of women of reproductive age (15-49 years) suffer from malaria-related morbidities [12]. Though there are recent Knowledge, Attitudes, and Practices (KAP) studies on malaria in Ghana, most are limited to district and regional level data [13–15]. In reviewing national level studies on malaria morbidity in Ghana, we found only recent reports from implementing partners of the 2016 GMIS. No study was found to have applied the 2016 GMIS data to examine knowledge and treatment practices among a specific population group such as women of reproductive age. The 2016 GMIS serves as a useful database to further understand the clinical and nonclinical aspects of malaria and address its morbidity concerns in Ghana. To scale up efforts towards malaria eradication, a heightened awareness of knowledge and treatment practices among varied subpopulation groups in Ghana is relevant, an identified gap that this study seeks to close in Ghana. The study examined reproductive-age women's knowledge and care seeking for malaria prevention and control in Ghana. Since reproductive-age women's productive lives affect other household member health needs [16], addressing reproductive-age knowledge and care seeking for malaria prevention is important to the overall promotion of health and well-being at the family level. Policy and future study suggestions presented in this study are useful for improving control strategies in Ghana and in malaria endemic areas in other countries in Sub-Saharan Africa (SSA).

## 2. Materials and Methods

### 2.1. Data

Data from the 2016 GMIS were analysed. The survey was implemented by partners made up of Ghana Statistical Service (GSS), National Public Health Reference Laboratory (NPHRL), DHS Program, PATH-MACEPA, the Malaria Consortium, Center for Disease Control (CDC), and the Carter Centre. The survey collected data on the epidemiology of malaria and household behaviours regarding malaria prevention and treatment in Ghana. Information collected included ownership and use of Insecticide Treated Nets (ITNs), Indoor Residual Spraying (IRS) with insecticides, treatments for fevers in children under 5 years of age, malaria and its prevention among pregnant women, and biomarker test for anaemia and malaria among pregnant women and children. For the purpose of this study, knowledge and care seeking related variables on malaria among reproductive-age women (15-49 years) were analysed.

### 2.2. Independent Variables

We included twelve (12) independent variables, after partial least squares regression (PLS) analysis was conducted to remove any variables that may have correlated effects in the final regression. Independent variables included in the final model were age, wealth index, spatial location (region), place of residence, ownership of bed net, sleep under mosquito bed, pregnancy status, type of mosquito bed net(s) slept under which were used in the analysis as presented in the original data file. Educational status was recategorized into three levels: no education, primary, and secondary, while religion was recategorized into 4 responses: Christianity, Islam, Traditional, and no religion. Parity status was recategorized into 4 responses: nulliparous 1-2 births, 3-4 births, and 5+ births. Type of toilet facility at household type was recategorized into 4 groups (flush toilet, pit latrine, open defecation, bucket type, and not a Dejure).

### 2.3. Dependent Variables

Two main outcome dichotomous variables were assessed as presented in Table 1: (1) overall knowledge of malaria causes, signs, symptoms, and prevention and (2) care-seeking practice assessed by respondent's awareness that malaria is covered by health insurance. In order to get an index variable to represent each level of the outcome measure on knowledge (causes, signs, symptoms, and prevention), mean scores were estimated for knowledge of causes, signs, and symptom/prevention. All mean scores below average mean score were considered poor knowledge while means scores from mid-point upwards as good knowledge (see Table 2). Care-seeking practice was assessed using the single variable, awareness that malaria is covered by health insurance.

### 2.4. Statistical Analysis

Descriptive profile of respondents was determined first, followed by multilevel mixed-effects logistic regression model to assess binary or binomial responses with suppress constant term from the fixed-effects equation. Multilevel logistic models (MLLM) were used to examine the influence of the effect of independent variables on dependent variables, using suppress constant term from the random-effects equation and restricted maximum likelihood estimation with displaying constraints.

MLLM was appropriate for this research due to the cluster sampling design adopted in the MIS data collection process. The MLLM allows one to account for the clustering of socioeconomic characteristics within clusters of higher-level units when estimating the effect of subject and cluster characteristics on subject outcomes [17] which in our case is knowledge of causes, signs and symptoms, and prevention of malaria. Stata version 14 was used to analyse all data. Statistical significance was considered at *α*=0.05.

## 3. Results

### 3.1. Sociodemographics of Respondents

A total of 5,150 women were surveyed during the 2016 MIS, 964 participants (19%) were within 15-19 years, 880 (17%) within 25-29 years, and 419 (8%) within 45-49 years. On wealth status, 1440 participants (28%) were in the poorest wealth quintile, 916 participants (18%) were within the middle wealth index, and 912 participants (18%) were in the richest wealth index. The Northern region recorded the highest (12%) while Upper West region recorded the lowest (9%) of survey respondents. More than half (57%) of the respondents were educated above secondary school level with 25% without any formal education. More than half of respondents (54%) lived in rural areas. Nulliparous women constituted 30% of respondents, while multiparas (3+births) women constituted 40.8%. The correlation coefficients of ever-experience for malaria, knowledge of malaria, knowledge of signs and symptoms, and knowledge of protection of malaria are presented in Table 3.

### 3.2. Knowledge of Malaria Causes and Preventions

About 6 out of 10 reproductive-aged women were aware ITNs can prevent malaria (see Table 4). However, only 47% slept under ITN the night prior to survey. About half (51%) of the respondents reported the use of pit latrines as toilet type at the household level. Open defecation was practiced by 27% of the sampled population. Regarding knowledge of causes of malaria, 37% were found to have poor knowledge while 62% had good knowledge. About 8 out of 10 women, however, had good knowledge of signs and symptoms of malaria. About 35.4% had poor knowledge while 65% had good knowledge of prevention practices. Overall, 60% of respondents registered as members of the national health insurance scheme.

### 3.3. Associations regarding Knowledge of Causes of Malaria among Respondents (Adjusted)

Table 5 presents results of knowledge of causes of malaria and their associations among respondents. Relative to women aged 15-19 years, women aged 20-24 and 30-49 years were significantly associated with women with good knowledge of the causes of malaria. Women in richer wealth quartile were significantly associated with women with good knowledge of causes of malaria, compared to women in richest wealth quartile. Women in 6 regions (Western, Central, Volta, Northern Upper East, and Upper West) were significantly associated with those with good knowledge of causes of malaria, compared to women in the Greater Accra region. Women with no education had a significant association with good knowledge of causes of malaria compared to women educated to secondary and above level. Women in rural areas had a significant association with good knowledge of causes of malaria, compared to urban women. Surprisingly, noneducated women were significantly associated with women with good knowledge of causes of malaria, compared with women educated at secondary level.

### 3.4. Associations regarding Knowledge of Signs and Symptoms of Malaria

Age, wealth index, region, educational status, place of residence, household toilet type, and religion were significantly associated with women with good knowledge of signs and symptoms of malaria (see Table 6, model 2). Increasing age showed increasing odds on knowledge of signs and symptoms of malaria as presented in model 2. Women aged 35-39 years [aOR=2.23, 95% CI: 1.68-2.95], 40-44 years [aOR=2.16, 95% CI: 1.59-2.89], and 45-49 years [aOR=2.34, 95% CI: 1.68-3.24] were more likely to have good knowledge of the signs and symptoms of malaria compared to women aged 15-19 years. Increasing wealth also showed increased odds for knowledge of signs and symptoms. However, women in poorest and poorer wealth groups were significantly associated with women with good knowledge of signs and symptoms, compared to women in richest wealth group. Women from Western, Ashanti, Northern, Upper East, and Upper West regions were significantly associated with women with good knowledge of signs and symptoms of malaria, compared to women in the Greater Accra region.

Across regions, women in the Volta and Northern regions had significant and high odds for good knowledge of signs and symptoms, compared to women in the Greater Accra region (aOR=2.70, 95% CI 0.81-4.04) and (aOR=2.09, 95% CI 1.41-3.11), respectively. Uneducated women and those educated to the primary level were significantly associated with women with good knowledge of signs and symptoms of malaria. Women who belong to traditional and other religious affiliations were also significantly associated with women with good knowledge of signs and symptoms, compared to women who belong to the Islamic religion.

### 3.5. Associations regarding Malaria Prevention

Women aged 20-39 years were significantly associated with women with good knowledge of malaria prevention, compared with women aged 15-19 years in regression model 2. Increasing wealth showed increasing odds regarding good knowledge of malaria prevention. In Table 7 (model 1), women in richer wealth index were significantly associated with those with good knowledge of prevention of malaria, compared to women in the richest wealth index. However, in model 2 (adjusted), women in the richer wealth index were not significant regarding good knowledge of malaria prevention. Women in 2 regions (Western and Volta) were significantly associated with women with good knowledge of malaria prevention, compared to women in the Greater Accra region. Relative to women in urban areas, women in rural areas were significantly associated with those with good knowledge of malaria prevention. Women who belong to other religious status were significantly associated with those with good knowledge, relative to women who belong to Islamic religion. Uneducated women and those educated at primary level were significantly associated with women with good knowledge of malaria prevention, compared to higher educated women.

### 3.6. Associations regarding Care Seeking for Malaria Treatments

Women in 7 regions (Western, Volta, Eastern, Brong Ahafo, Northern, Upper East, and Upper West) showed significant association with women who knew malaria treatment was covered under national health insurance, compared to women in the Greater Accra region** (**see Table 8). Women in the Upper East and Brong Ahafo regions had the highest odds of women who knew that malaria treatments were covered under health insurance, compared to women in the Greater Accra region (aOR=3.73, 95% CI 2.55-5.44) and (aOR=3.41, 95% CI 2.43-4.79), respectively. Relative to women in urban areas, women in rural areas were significantly associated with women who knew malaria treatment was covered under the national health insurance scheme. Nulliparous women were significantly associated with women who knew malaria treatment was covered under national health insurance scheme, compared to women with parity level of 5 and above. Relative to women who slept under mosquito net, women who did not sleep under any mosquito net were significantly associated with those with knowledge that malaria treatments are covered under the national health insurance scheme.

## 4. Discussion

Reproductive-age women had good knowledge associated with the causes, signs/symptoms, and prevention of malaria. This study found a 15% differential among ITNs awareness and use among reproductive-age women Ghana. This indicates an awareness-practice gap for malaria control among reproductive-age women in Ghana, which has long been documented in other studies [18, 19]. Similarly, our finding is consistent with the 2016 GMIS report that documented a 66% of household population in Ghana having access to Long Lasting Insecticide Net (LLIN) with only 42% of households sleeping under a net the night before the survey [12]. Novel approaches for easy adoption and use of ITNs need to be piloted in future funded studies to generate evidence on scaling up ITNs use in Ghana.

In Ghana, contextual demographic factors have been found to influence the knowledge-practice nexus regarding malaria control and prevention, with households with children under 5 years and pregnant women likely to have positive attitudes and practices towards malaria prevention [14]. Community level misconceptions on malaria control strategies and beliefs affect ITN use in Ghana [20, 21]. Behaviour change communication (BCC) strategies that address community level misconceptions and social beliefs on ITNs use could improve use among all population groups in Ghana. Studies in Ethiopia, Nigeria, Tanzania, Zambia, and Zimbabwe [22–26] found community and individual level factors that influence knowledge-practices differences, as found in Ghana.

### 4.1. Causes, Signs/Symptoms, Prevention, and Care Seeking for Malaria

Age, wealth and educational status, religion, region, place of residence (rural), and sleeping under ITN were found to significantly influence reproductive-age women's knowledge levels regarding causes, signs/symptoms, prevention, and care-seeking practices for malaria. The study finding reflects existing evidence in Ghana [27, 28], other African countries [29, 30], and developing countries [31] of the role of individual, social, environmental, and health system determinants of malaria among varied population groups. Nonuse of ITN was found to be statistically significant and associated with poor knowledge related to care seeking among reproductive-age women in Ghana. Specifically, it was found that women who did not sleep under mosquito bed net have poor knowledge of care seeking in the study. Malaria messaging targeting particular poor behavioural attitudes in Ghana may seem low, explaining why only 41% of women (15-49 years) heard of malaria messaging in the last 6 months prior to the survey [12]. At the policy level, more targeted education programs that seek to sustain behaviour practices need to be stepped up so that health facilities and district environmental health officers can help improve women and other caregiver's knowledge levels on malaria prevention and care seeking.

This study further posits that knowledge of the causes, signs/symptoms, and prevention of malaria among women aged 35 and older is high among reproductive-age women in Ghana. The findings show that increasing age (≥35 years) of a woman increases her knowledge levels on the causes, signs/symptoms, prevention, and care seeking for malaria, compared to young women. While this study did not draw causal links between older women knowledge and how these translate into malaria prevention strategies at the individual level, it can be posited that older women who have had previous malaria episodes have a high likelihood to know about causes and prevention strategies. Older women might have had several exposures to malaria messaging compared to younger women. It should be noted that this finding does not mean older women will necessarily translate knowledge to improved health behaviour since another study in Ghana found nondoers were more knowledgeable about malaria than doers [32].

Socioeconomic status (SES) measured through wealth status (poorest and poorer) and low education (no education) had undesirable outcomes regarding knowledge of signs/symptoms and prevention/coping mechanisms for malaria. Women in poorest and poorer wealth indices and those with no formal education were less likely to have knowledge regarding the signs and symptoms as well as malaria prevention compared to those in richest wealth group. In contrast, women with high wealth status (richer wealth quartile) were found to have a 125% likelihood of improved knowledge levels of the causes of malaria among reproductive-age women. Effects of poverty on poor knowledge of malaria from this study embody long held propositions that refer to malaria as a disease of poverty [33, 34]. However, other factors beyond poverty and socioeconomic status such as educational effects for early care seeking have been documented as vital in offsetting poverty and malaria care seeking among children in Gambia [35].

This study also found that significant regional differences exist regarding knowledge of causes, signs/symptoms, and prevention in Ghana. Regional variation of malaria among children in Ghana is reported [36], but not among reproductive-age women. Reproductive-age women in 3 regions: Ashanti, Brong Ahafo, and Eastern regions were observed to have the highest odds of association regarding poor knowledge of the causes of malaria. Reproductive-age women in Western Region were observed to have the lowest odds of association for poor knowledge of causes for malaria. The Volta and Northern regions had high odds of association regarding poor knowledge of signs/symptoms of malaria among reproductive-age women. Regional differences of knowledge-related measures among pregnant women and at the household were also reported in the 2016 GMIS report [12]. In addition, poor knowledge levels on causes and prevention of malaria were significantly associated with women who lived in rural areas. Despite that more than half of the population surveyed in the 2016 MIS was rural, majority of them were more likely to have good knowledge of care seeking with regard to malaria.

Nonawareness of malaria treatment covered by national health insurance was influenced by region, place of residence, and sleeping under a mosquito net. Reproductive-age women in four regions (Volta, Brong Ahafo, Upper East, and Upper West) had higher odds (>3) of not being aware malaria treatment was covered under health insurance. Additionally, rural women had higher odds of being unaware malaria treatment is covered by national health insurance. We also found reproductive-age women who did not sleep under a mosquito net had lesser odds of not being aware national health insurance covers malaria treatment.

The study has some limitations. Data used for analysis in this study is secondary and cross sectional, leaving little chance for assuming causality of associations. Secondly, malaria aetiology and outcomes may be influenced by other several factors (individual, environmental, and health system) which may not have been present however in the dataset, thus excluded from our analysis. The data is however a national representative and hence provides strong evidence of context factors that shape reproductive-age women views on malaria prevention and care seeking. The 2016 GMIS presented results at household levels for ITNs use and knowledge of causes of malaria based on individual variables. This study however estimated knowledge of outcomes based on index created from a cluster of variables. Methodologically, the importance of composite variable index to explain variations in health has been documented [37]; hence, the use of composite index in assessing knowledge related outcomes for malaria is useful.

## 5. Conclusions

This study found that age, wealth and educational status, religion, region, and place of residence (rural) significantly influence reproductive-age women knowledge levels regarding causes, signs/symptoms, and prevention of malaria. Older women (≥35 years) had higher likelihood of good knowledge levels of the causes, signs/symptoms, and prevention of malaria, compared to young women. Regional variations exist on knowledge levels and care seeking for malaria, with rural residents more likely to have poor knowledge and care seeking for malaria. Our study also observed a 15% differential among ITNs awareness and use among reproductive-age women Ghana. Thus, there is a need to intensify health education on malaria and the benefits of the use of mosquito nets at all levels. Furthermore, free distribution of mosquito nets should be scaled up to attain higher coverages. In addition, a collaborative effort among partners including the use of new technologies to vector imaging could help scale up efforts to improve knowledge and care seeking for malaria prevention and control in Ghana.

## Figures and Tables

**Table 1:** Index variables and their descriptions from data file.

Index variables	Variables description
Knowledge of causes of malaria	Thirteen (13) dichotomous variables with responses on eating and work habits, personal and environmental hygiene, bites from mosquitos, houseflies and hereditary factors, and other causes were used. Average scores were created on this item.

Knowledge of signs and symptoms	Ten (10) dichotomous variables that assessed respondents' views on body fevers, vomiting, appetite, body weakness, cough/chills, urine colour, bitterness in mouth and other signs and symptoms. An ordinal scale was created to assess good and poor knowledge.

Knowledge of prevention/protection from malaria	Eleven (11) dichotomous variables that collected information on respondents use of ITNs, use of mosquito repellents and sprays, personal and environmental hygiene, use of protective cloths, and household strategies to prevent mosquito bites. An ordinal scale was created to measure good and poor knowledge.

Treatment awareness under NHIS	A single dichotomous variable that measured respondent's awareness that treatment for malaria was covered under national health insurance.

Source: extracted from the GMIS 2016 data file for women.

**Table 2:** Grading scores for good and poor knowledge.

Level	Knowledge assessment scores		
	Cause	Signs and Symptoms	Protection
	Score=0-6	Score=0-9	Score=0-7
Poor	0-1	0-2	0-1
Good	2-5	3-9	2-7
Mean (SD)	1.97(0.93)	2.5(1.16)	2.01(0.96)

**Table 3:** Correlation of ever-experience of malaria, knowledge of causes, signs, and symptoms and protection of malaria.

Variable	EM	KCM	KSSM	KPM
EM	1			
KCM	0.061	1		
KSSM	0.1285	0.3067	1	
KPM	0.0527	0.4528	0.3832	1

Note: EM=episodes of malaria; KCM=knowledge of causes of malaria; KSSM= knowledge of signs and symptoms of malaria; KPM= knowledge of protection of malaria.

**Table 4:** Sociodemographics and descriptive results of Ghanaian reproductive women, 2016 Ghana Malaria Indicator Survey.

Demographic variable	Frequency	Percentage	Demographic variable	Frequency	Percentage
	N=5150	%		N=5150	%
*Age in 5-year groups*			*Slept under ITNs night prior to survey*		
15-19	964	18.7	No	2,727	53.0
20-24	844	16.4	Yes	2,423	47.1
25-29	880	17.1	*Currently pregnant*		
30-34	799	15.5	No/unsure	4,799	93.2
35-39	675	13.1	Yes	351	6.8
40-44	569	11.1	*Type of mosquito bed net(s) slept under*		
45-49	419	8.1	No net	2,727	53.0
*Wealth index*			Treated net	2,384	46.3
Poorest	1,440	28.0	Both treated and untreated nets	1	0.0
Poorer	946	18.4	Only untreated nets	38	0.7
Middle	916	17.8	*Type of toilet facility*		
Richer	936	18.2	Flush toilet	1,030	20.0
Richest	912	17.7	Pit latrine	2,623	50.9
*Region*			Open defecation	1,371	26.6
Western	451	8.8	Bucket toilet	11	0.2
Central	502	9.8	Not a Dejure	115	2.2
Greater Accra	560	10.9	*Age of household head in groups*		
Volta	553	10.7	Below 20	18	0.4
Eastern	465	9.0	20-29	639	12.4
Ashanti	563	10.9	30-39	1,323	25.7
Brong Ahafo	500	9.7	40-49	1,448	28.1
Northern	591	11.5	50-59	931	18.1
Upper east	528	10.3	60-69	434	8.4
Upper west	437	8.5	70+	318	6.2
*Educational level*			Don't know	39	0.8
No education	1,283	24.9	*Covered by health insurance*		
Primary	918	17.8	No	2,057	39.9
Secondary	2,949	57.3	Yes	3,093	60.1
*Religion*			*Aware that malaria is covered under the NHIS*		
Christianity	3,774	73.3	No	1,049	20.4
Islam	1,147	22.3	Yes	4,101	79.6
Traditional/spiritualist	128	2.5			
No religion	101	2.0	*Knowledge on causes of Malaria*		
*Place of residence*			Poor knowledge	1939	37.7
Urban	2,369	46.0	Good Knowledge	3211	62.3
Rural	2,781	54.0			
*Parity (living children)*			*Knowledge on signs and symptoms of Malaria*		
Nulliparous	1,540	29.9	Poor knowledge	965	18.7
1-2	1,510	29.3	Good Knowledge	4185	81.3
3-4	1,225	23.8			
5+	875	17.0			
*Have mosquito bed net*			*Knowledge on protection of Malaria*		
No	883	17.2	*Poor knowledge*	**1822**	**35.4**
Yes	4,267	82.9	*Good Knowledge*	**3328**	**64.6**

**Table 5:** Sociodemographics and knowledge of causes of malaria associations among reproductive-age women, 2016 Ghana Malaria Indicator Survey.

Demographics	Knowledge of cause of malaria f (%)			COR (95 C.I) p-value	AOR(95 C.I)p-value
	Poor knowledge=0 (n=1939)	Good knowledge =1 (n=3211)	Total=5150	Model 1	Model 2
*Age in 5-year groups*					
15-19	408(21.0)	556(17.3)	964(18.7)	Ref	Ref
20-24	315(16.3)	529(16.4)	844(16.4)	1.23(1.02-1.49)*∗*	1.23(1.01-1.50)*∗*
25-29	350(18.1)	530(16.6)	880(17.1)	1.11(0.92-1.34)	1.14(0.94-1.38)
30-34	296(15.3)	503(15.7)	799(15.5)	1.25(1.02-1.51)*∗*	1.37(1.12-1.67)*∗∗*
35-39	225(11.6)	450(14.0)	675(13.1)	1.47(1.20-1.80)*∗∗∗*	1.69(1.35-2.11)*∗∗∗*
40-44	198(10.2)	371(11.6)	569(11.1)	1.37(1.10-1.70)*∗∗*	1.57(1.24-1.98)*∗∗∗*
45-49	147(7.6)	272(8.5)	419(8.1)	1.36(1.07-1.72)*∗*	1.57(1.22-2.03)*∗∗∗*

*Wealth index*					
Poorest	659(34.0)	781(24.3)	1440(28.0)	0.61(0.51-0.72)*∗∗∗*	1.04(0.78-1.37)
Poorer	372(19.2)	574(17.9)	946(18.4)	0.79(0.66-0.96)*∗*	1.13(0.88-1.45)
Middle	314(16.2)	602(18.8)	916(17.8)	0.99(0.81-1.19)	1.16(0.92-1.47)
Richer	284(14.7)	652(20.3)	936(18.2)	1.18(0.97-1.44)	1.25(1.01-1.55)*∗*
Richest	310(16.0)	602(18.8)	912(17.7)	Ref	Ref

*Region*					
Western	216(11.1)	235(7.3)	451(8.8)	0.38(0.29-0.49)*∗∗∗*	0.41(0.31-0.54)*∗∗∗*
Central	194(10.0)	308(9.6)	502(9.8)	0.55(0.42-0.71)*∗∗∗*	0.59(0.44-0.77)*∗∗∗*
Greater Accra	144(7.4)	416(13.0)	560(10.9)	Ref	Ref
Volta	256(13.2)	297(9.3)	553(10.7)	0.40(0.31-0.52)*∗∗∗*	0.44(0.34-0.59)*∗∗∗*
Eastern	140(7.2)	325(10.1)	465(9.0)	0.80(0.61-1.06)	0.81(0.61-1.09)
Ashanti	153(7.9)	410(12.8)	563(10.9)	0.93(0.71-1.21)	0.93(0.71-1.22)
Brong Ahafo	155(8.0)	345(10.7)	500(9.7)	0.77(0.59-1.01)	0.84(0.63-1.11)
Northern	255(13.2)	336(10.5)	591(11.5)	0.46(0.36-0.59)*∗∗∗*	0.67(0.50-0.91)*∗*
Upper east	222(11.5)	306(9.5)	528(10.3)	0.48(0.37-0.62)*∗∗∗*	0.68(0.50-0.93)*∗*
Upper west	204(10.5)	233(7.3)	437(8.5)	0.40(0.31-0.52)*∗∗∗*	0.54(0.40-0.74)*∗∗∗*
*Educational level*					
No education	595(30.7)	688(21.4)	1283(24.9)	0.60(0.52-0.68)*∗∗∗*	0.67(0.55-0.79)*∗∗∗*
Primary	340(17.5)	578(18.0)	918(17.8)	0.88(0.75-1.02)	0.92(0.78-1.08)
Secondary	1004(51.8)	1945(60.6)	2949(57.3)	Ref	Ref

*Place of residence*					
Urban	739(38.1)	1630(50.8)	2369(46.0)	Ref	Ref
Rural	1200(61.9)	1581(49.2)	2781(54.0)	0.60(0.53-0.67)*∗∗∗*	0.77(0.65-0.89)*∗∗∗*

*Slept under mosquito bed net*					
No	973(50.2)	1754(54.6)	2727(53.0)	1.20(1.07-1.34)*∗∗*	1.04(0.91-0.17)
Yes	966(49.8)	1457(45.4)	2423(47.1)	Ref	Ref

*∗*P < 0.05, *∗∗*p < 0.01, *∗∗∗*p < 0.001. Ref: denotes reference group.

**Table 6:** Sociodemographics and knowledge of signs and symptoms associations among reproductive-age women, 2016 Ghana Malaria Indicator Survey.

Demographics	Knowledge of signs and symptoms f (%)			COR (95 C.I) p-value	AOR (95 C.I) p-value
	Poor knowledge=0 (n==965)	Good knowledge=1 (n=4185)	Total=5150	Model 1	Model 2
*Age in 5-year groups*					
15-19	230(23.8)	734(17.5)	964(18.7)	Ref	Ref
20-24	163(16.9)	681(16.3)	844(16.4)	1.31(1.04-1.64)*∗*	1.35(1.04-1.72)*∗*
25-29	151(15.7)	729(17.4)	880(17.1)	1.51(1.20-1.90)*∗∗∗*	1.66(1.27-2.13)*∗∗∗*
30-34	140(14.5)	659(15.8)	799(15.5)	1.47(1.17-1.87) *∗∗∗*	1.84(1.41-2.41)*∗∗∗*
35-39	113(11.7)	562(13.4)	675(13.1)	1.56(1.21-2.00) *∗∗∗*	2.23(1.68-2.95)*∗∗∗*
40-44	98(10.2)	471(11.3)	569(11.1)	1.51(1.16-1.96)*∗∗*	2.16(1.59-2.89)*∗∗∗*
45-49	70(7.3)	349(8.3)	419(8.1)	1.56(1.16-2.10)*∗∗*	2.34(1.68-3.24)*∗∗∗*

*Wealth index*					
Poorest	374(38.8)	1066(25.5)	1440(28.0)	0.30(0.24-0.39) *∗∗∗*	0.49(0.34-0.72) *∗∗∗*
Poorer	216(22.4)	730(17.4)	946(18.4)	0.36(0.28-0.47) *∗∗∗*	0.54(0.38-0.76) *∗∗∗*
Middle	139(14.4)	777(18.6)	916(17.8)	0.60(0.45-0.79) *∗∗∗*	0.76(0.54-1.06)
Richer	148(15.3)	788(18.8)	936(18.2)	0.57(0.43-0.75) *∗∗∗*	0.66(0.48-0.89)*∗*
Richest	88(9.1)	824(19.7)	912(17.7)	Ref	Ref

*Region*					
Western	149(15.4)	302(7.2)	451(8.8)	0.34(0.25-0.46) *∗∗∗*	0.47(0.34-0.67) *∗∗∗*
Central	111(11.5)	391(9.3)	502(9.8)	0.59(0.43-0.81) *∗∗∗*	0.90(0.63-1.29)
Greater Accra	80(8.3)	480(11.5)	560(10.9)	Ref	Ref
Volta	58(6.0)	495(11.8)	553(10.7)	1.42(0.99-2.04)	2.70(0.81-4.04)
Eastern	83(8.6)	382(9.1)	465(9.0)	0.77(0.55-1.07)	1.07(0.74-1.55)
Ashanti	63(6.5)	500(12.0)	563(10.9)	1.32(0.93-1.88)	1.54(1.06-2.23)*∗*
Brong Ahafo	80(8.3)	420(10.0)	500(9.7)	0.88(0.63-1.22)	1.43(0.99-2.07)
Northern	120(12.4)	471(11.3)	591(11.5)	0.65(0.48-0.89)*∗*	2.09(1.41-3.11) *∗∗∗*
Upper east	118(12.2)	410(9.8)	528(10.3)	0.58(0.42-0.79*∗∗∗*	1.72(1.16-2.56)*∗*
Upper west	103(10.7)	334(8.0)	437(8.5)	0.54(0.39-0.75) *∗∗∗*	1.50(1.01-2.23)*∗*

*Educational level*					
No education	358(37.1)	925(22.1)	1283(24.9)	0.41(0.35-0.49) *∗∗∗*	0.41(0.33-0.51) *∗∗∗*
Primary	199(20.6)	719(17.2)	918(17.8)	0.58(0.48-0.70) *∗∗∗*	0.62(0.50-0.76) *∗∗∗*
Secondary	408(42.3)	2541(60.7)	2949(57.3)	Ref	Ref

*Religion*					
Christian	656(68.0)	3118(74.5)	3774(73.3)	1.13(0.96-1.34)	1.01(0.82-1.22)
Islam	221(22.9)	926(22.1)	1147(22.3)	Ref	Ref
Traditional/spiritualist	41(4.3)	87(2.1)	128(2.5)	0.51(0.34-0.75) *∗∗∗*	0.57(0.38-0.87)*∗*
Other religions	47(4.9)	54(1.3)	101(2.0)	0.27(0.18-0.42) *∗∗∗*	0.31(0.21-0.59) *∗∗∗*

*Place of residence*					
Urban	327(33.9)	2042(48.8)	2369(46.0)	Ref	Ref
Rural	638(66.1)	2143(51.2)	2781(54.0)	0.54(0.46-0.62) *∗∗∗*	0.89(0.73-1.08)

*Currently pregnant*					
No	885(91.7)	3914(93.5)	4799(93.2)	1.31(1.01-1.69)*∗*	1.26(0.96-1.67)
Yes	80(8.3)	271(6.5)	351(6.8)	Ref	Ref

*∗*P < 0.05, *∗∗*p < 0.01, *∗∗∗*p < 0.001. Ref: denotes reference group.

**Table 7:** Sociodemographics and prevention of malaria associations among reproductive-age women, 2016 Ghana Malaria Indicator Survey.

Demographics	Knowledge of malaria prevention f (%)			COR (95 C.I) p-value	AOR (95 C.I) p-value
	Poor knowledge (n=1822)	Good knowledge (n=3328)	Total=5150	Model 1	Model 2
*Age in 5-year groups*					
15-19	352(19.3)	612(18.4)	964(18.7)	Ref	Ref
20-24	256(14.1)	588(17.7)	844(16.4)	1.32(1.09-1.61)*∗∗*	1.44(1.15-1.81)*∗∗*
25-29	298(16.4)	582(17.5)	880(17.1)	1.12(0.93-1.36)	1.32(1.03-1.68)*∗*
30-34	288(15.8)	511(15.4)	799(15.5)	1.02(0.84-1.24)	1.33(1.01-1.75)*∗*
35-39	241(13.2)	434(13.0)	675(13.1)	1.04(0.84-1.27)	1.50(1.12-2.01)*∗*
40-44	220(12.1)	349(10.5)	569(11.1)	0.91(0.74-1.13)	1.30(0.96-1.77)
45-49	167(9.2)	252(7.6)	419(8.1)	0.87(0.69-1.10)	1.30(0.94-1.81)

*Wealth index*					
Poorest	711(39.0)	729(21.9)	1440(28.0)	0.27(0.22-0.32) *∗∗∗*	0.49(0.37-0.67) *∗∗∗*
Poorer	372(20.4)	574(17.3)	946(18.4)	0.40(0.33-0.50) *∗∗∗*	0.70(0.53-0.92)*∗∗*
Middle	308(16.9)	608(18.3)	916(17.8)	0.52(0.42-0.64) *∗∗∗*	0.74(0.57-0.95)*∗*
Richer	242(13.3)	694(20.9)	936(18.2)	0.75(0.60-0.93)*∗∗*	0.93(0.73-1.17)
Richest	189(10.4)	723(21.7)	912(17.7)	Ref	Ref

*Region*					
Western	184(10.1)	267(8.0)	451(8.8)	0.39(0.29-0.51) *∗∗∗*	0.56(0.41-0.75) *∗∗∗*
Central	172(9.4)	330(9.9)	502(9.8)	0.51(0.39-0.67) *∗∗∗*	0.79(0.59-1.07)
Greater Accra	118(6.5)	442(13.3)	560(10.9)	Ref	Ref
Volta	277(15.2)	276(8.3)	553(10.7)	0.27(0.20-0.35) *∗∗∗*	0.42(0.32-0.56) *∗∗∗*
Eastern	119(6.5)	346(10.4)	465(9.0)	0.78(0.58-1.04)	0.15(0.84-1.57)
Ashanti	148(8.1)	415(12.5)	563(10.9)	0.75(0.57-0.99)*∗*	0.88(0.66-1.18)
Brong Ahafo	148(8.1)	352(10.6)	500(9.7)	0.63(0.48-0.84) *∗∗∗*	1.02(0.75-1.38)
Northern	250(13.7)	341(10.3)	591(11.5)	0.36(0.28-0.47) *∗∗∗*	1.11(0.80-1.53)
Upper east	211(11.6)	317(9.5)	528(10.3)	0.40(0.31-0.52) *∗∗∗*	1.17(0.84-1.62)
Upper west	195(10.7)	242(7.3)	437(8.5)	0.33(0.25-0.44) *∗∗∗*	0.93(0.67-1.29)

*Educational level*					
No education	677(37.2)	606(18.2)	1283(24.9)	0.32(0.28-0.36) *∗∗∗*	0.43(0.35-0.51) *∗∗∗*
Primary	373(20.5)	545(16.4)	918(17.8)	0.52(0.44-0.61) *∗∗∗*	0.63(0.53-0.74) *∗∗∗*
Secondary	772(42.4)	2177(65.4)	2949(57.3)	Ref	Ref

*Religion*					
Christian	1236(67.8)	2538(76.3)	3774(73.3)	1.36(1.20-1.56) *∗∗∗*	1.14(0.97-1.34)
Islam	458(25.1)	689(20.7)	1147(22.3)	Ref	Ref
Traditional/spiritualist	66(3.6)	62(1.9)	128(2.5)	0.62(0.43-0.90)	1.22(0.83-1.79)
Other religions	62(3.4)	39(1.2)	101(2.0)	0.42(0.28-0.63) *∗∗∗*	0.55(0.35-0.86)*∗∗*

*Place of residence*					
Urban	609(33.4)	1760(52.9)	2369(46.0)	Ref	Ref
Rural	1213(66.6)	1568(47.1)	2781(54.0)	0.45(0.40-0.50) *∗∗∗*	0.79(0.67-0.92)*∗∗*

*Living Children*					
No child	460(25.3)	1080(32.5)	1540(29.9)	1.92(1.62-2.28) *∗∗∗*	1.18(0.89-1.58)
1-2	511(28.1)	999(30.0)	1510(29.3)	1.60(1.35-1.90) *∗∗∗*	0.97(0.77-1.22)
3-4	457(25.1)	768(23.1)	1225(23.8)	1.38(1.15-1.64) *∗∗∗*	1.03(0.84-1.25)
5+	394(21.6)	481(14.5)	875(17.0)	Ref	Ref

*∗*P < 0.05, *∗∗*p < 0.01, *∗∗∗*p < 0.001. Ref: denotes reference group.

**Table 8:** Sociodemographics and malaria treatments by health insurance associations among reproductive-age women, 2016 Ghana Malaria Indicator Survey.

Demographics	Aware that malaria is covered by insurance f (%)			COR (95 C.I) p-value	AOR (95 C.I) p-value
	No=1049	Yes=4101	Total=5150	Model 1	Model 2
	N (%)	N (%)	N (%)		
*Wealth index*					
Poorest	226(21.5)	1214(29.6)	1440(28.0)	1.76(1.43-2.16) *∗∗∗*	0.92(0.66-1.27)
Poorer	169(16.1)	777(19.0)	946(18.4)	1.51(1.20-1.88) *∗∗∗*	1.02(0.76-1.35)
Middle	190(18.1)	726(17.7)	916(17.8)	1.25(1.00-1.56)	0.99(0.76-1.28)
Richer	239(22.8)	697(17.0)	936(18.2)	0.96(0.77-1.18)	0.89(0.71-1.13)
Richest	225(21.5)	687(16.8)	912(17.7)	Ref	Ref

*Region*					
Western	105(10.0)	346(8.4)	451(8.8)	1.71(1.29-2.26) *∗∗∗*	1.46(1.08-1.97)*∗∗*
Central	146(13.9)	356(8.7)	502(9.8)	1.26(0.97-1.63)	1.05(0.78-1.39)
Greater Accra	191(18.2)	369(9.0)	560(10.9)	Ref	Ref
Volta	65(6.2)	488(11.9)	553(10.7)	3.89(2.84-5.31) *∗∗∗*	3.57(2.55-4.99) *∗∗∗*
Eastern	98(9.3)	367(9.0)	465(9.0)	1.94(1.46-2.57) *∗∗∗*	1.66(1.23-2.24) *∗∗∗*
Ashanti	157(15.0)	406(9.9)	563(10.9)	1.34(1.03-1.72)	1.17(0.91-1.52)
Brong Ahafo	59(5.6)	441(10.8)	500(9.7)	3.87(2.80-5.34) *∗∗∗*	3.41(2.43-4.79) *∗∗∗*
Northern	96(9.2)	495(12.1)	591(11.5)	2.67(2.02-3.53) *∗∗∗*	2.98(2.11-4.19) *∗∗∗*
Upper east	69(6.6)	459(11.2)	528(10.3)	3.44(2.53-4.68) *∗∗∗*	3.73(2.55-5.44) *∗∗∗*
Upper west	63(6.0)	374(9.1)	437(8.5)	3.07(2.23-4.23) *∗∗∗*	3.12(2.15-4.53) *∗∗∗*

*Educational level*					
No education	231(22.0)	1052(25.7)	1283(24.9)	1.26(1.07-1.49)*∗∗*	0.82(0.66-1.01)
Primary	177(16.9)	741(18.1)	918(17.8)	1.16(0.97-1.40)	0.93(0.76-1.13)
Secondary	641(61.1)	2308(56.3)	2949(57.3)	Ref	Ref

*Place of residence*					
Urban	599(57.1)	1770(43.2)	2369(46.0)	Ref	Ref
Rural	450(42.9)	2331(56.8)	2781(54.0)	1.75(1.53-2.01) *∗∗∗*	1.44(1.21-1.74) *∗∗∗*

*Parity (living children)*					
Nulliparous	345 (32.9)	1195 (29.1)	1540 (29.9)	0.73(0.59-0.90)*∗∗*	0.78(0.61-1.00)*∗*
1-2	321(30.6)	1189 (29.0)	1510 (29.3)	0.78(0.63-0.96)*∗*	0.84(0.66-1.06)
3-4	231(22.0)	994 (24.2)	1225 (23.8)	0.90(0.72-1.13)	0.94(0.74-1.19)
5+	152 (14.5)	723 (17.6)	875 (17.0)	Ref	Ref

*Slept under mosquito bed net*					
No	663 (63.2)	2064 (50.3)	2727 (53.0)	0.59(0.51-0.68) *∗∗∗*	0.76(0.65-0.91) *∗∗∗*
Yes	386 (36.8)	2037 (49.7)	2423 (47.1)	Ref	Ref

*∗*P < 0.05, *∗∗*p < 0.01, *∗∗∗*p < 0.001. Ref: denotes reference group.

## Data Availability

The data and the analysis coding structure used to support the findings of this study are available from the corresponding author upon request.

## Abbreviation

### Abbreviations

CDC: Center for Disease Control
GMIS: Ghana Malaria Indicator Survey
GSS: Ghana Statistical Service
ITN: Insecticide Treated Nets
IRS: Indoor Residual Spraying
NPHRL: National Public Health Reference Laboratory
NMCP: National Malaria Control Programme
RDT: Rapid Diagnostic Tests
SSA: Sub-Saharan Africa
WHO: World Health Organization.
